# Biological Significance of Kisspeptin–Kiss 1 Receptor Signaling in the Habenula of Teleost Species

**DOI:** 10.3389/fendo.2018.00222

**Published:** 2018-05-07

**Authors:** Satoshi Ogawa, Ishwar S. Parhar

**Affiliations:** Brain Research Institute, Jeffrey Cheah School of Medicine and Health Sciences, Monash University Malaysia, Bandar Sunway, Malaysia

**Keywords:** kisspeptin 1, teleosts fish, zebrafish, non-hypothalamic, Gpr54/Kiss1r

## Abstract

Kisspeptin is a neuropeptide, encoded by kisspeptin 1 (KISS1)/Kiss1 gene, which primarily acts as the regulator of reproductive functions *via* its receptor, kisspeptin receptor (KissR) in vertebrates. In the brain, Kiss1 gene is mainly expressed in the hypothalamic region, but *KissR* gene is widely distributed throughout the brain, suggesting that kisspeptin–KissR system may be involved in not only reproductive, but also non-reproductive functions. In non-mammalian vertebrates, there are two or more kisspeptin and KissR types. The zebrafish (*Danio rerio*) possess two kisspeptin (Kiss1 and Kiss2) and their respective receptors [Kiss1 receptor (KissR1) and KissR2]. In the brain of zebrafish, while Kiss2 is expressed in the preoptic-hypothalamic area, Kiss1 is predominantly expressed in the habenula, an evolutionarily conserved epithalamic structure. Similarly, KissR1 is expressed only in the habenula, while KissR2 is widely distributed in the brain, suggesting that the two kisspeptin systems play specific roles in the brain. The habenular Kiss1 is involved in the modulation of the raphe nuclei and serotonin-related behaviors such as fear response in the zebrafish. This review summarizes the roles of multiple kisspeptin–KissR systems in reproductive and non-reproductive functions and neuronal mechanism, and debates the biological and evolutional significance of habenular kisspeptin–KissR systems in teleost species.

## Introduction

During the past decade, the field of reproductive neuroendocrinology has shifted from its major focus on the hypothalamus–pituitary–gonadal (HPG) axis comprising gonadotropin-releasing hormone (GnRH), gonadotropins [luteinizing hormone (LH) and follicle-stimulating hormone (FSH)], and gonadal steroids to the next level with the discovery of RFamides, which include kisspeptin and gonadotropin-inhibitory hormone (also known as LPXRFamide). Kisspeptin is a neuropeptide, encoded by kisspeptin 1 (KISS1)/Kiss1 gene which was originally identified as a metastasis suppressor gene (1). Kisspeptin binds to its receptor, kisspeptin receptor (KissR), which was originally identified as an orphan G-protein-coupled receptor-54 (GPR54, also known as hOT7T175) (2). Kisspeptin consists of core peptides, including 54-, 14-, and 13-amino acids peptides and its processed mature, biologically active 10-amino acid peptide (Kiss1-10). In 2003, two studies reported consecutively the role of kisspeptin–KissR signaling in reproduction, particularly the control of GnRH-LH secretion during the onset of puberty in mammals (3, 4). Since then, numerous studies have demonstrated the role of kisspeptin–KissR signaling, neuroanatomy, and neuro-molecular mechanisms underlying the control of reproductive physiology in mammalian species (5). We have been interested to understand the importance of kisspeptin–KissR signaling in the vertebrate reproduction from an evolutional perspective using non-mammalian vertebrates. In 2004, we were the first to identify the non-mammalian KissR-like sequence from a cichlid fish, Nile tilapia (*Oreochromis niloticus*) and also demonstrated their gene expression in GnRH neurons using a laser capture microdissection technique (6). As for kisspeptin in teleosts, fish Kiss1 gene was first reported in zebrafish (*Danio rerio*) (7, 8). Interestingly, we found another gene encoding kisspeptin-like structure, which is slightly different from Kiss1 in the zebrafish and medaka (*Oryzias latipes*), we, therefore, named it Kiss2 (9). Similar to kisspeptins in teleosts, there are two or more KissR types, which are distributed in different patterns in the brain (10), suggesting specific role for two kisspeptin types in fish brain. Although in the mammalian species, kisspeptin–KissR system primarily targets GnRH neurons, in fact, kisspeptin neurons actually send their projections to a large number of brain areas and KissR are widely distributed in the brain (11–13). In addition, Kiss1 gene is also expressed in some extra-hypothalamic regions, such as the hippocampal dentate gyrus (14) and the medial amygdala (15). Surprisingly, in the zebrafish and medaka, knockout of two kisspeptins (*kiss1* and *kiss2*) and KissRs [Kiss1 receptor (*kissr1)* and *kissr2*] genes had no obvious effect on their reproductive capability (16, 17). These observations indicate that kisspeptin–KissR system may play roles in processing several non-reproductive functions. In fact, a functional MRI study in humans has recently revealed that kisspeptin modulates limbic brain activity in response to sexual and emotional stimuli, and influences mood in healthy men (18). Given that the zebrafish model has clear distinct neuroanatomical patterns of two kisspeptin–KissR systems, the zebrafish is believed to be an ideal model to understand differential role and regulatory mechanism of the two kisspeptin–KissR systems. We have been particularly interested to understand the role of Kiss1 in the habenula. The habenula is an evolutionary conserved epithalamic structure, which is involved in certain forms of emotive decision making in primates. Recent discoveries in primates by Dr. Hikosaka’s group indicate that the habenula plays a prominent role in emotive behavioral choice through neuromodulation of the dopamine and the serotonin systems (19). In addition, the habenula is involved in behavioral responses to pain, stress, anxiety, sleep, and reward. The dysfunction of the habenula is associated with neurological problems, such as depression, schizophrenia, and drug-induced psychosis (20). Therefore, the habenula has been a recent focus as a potential therapeutic target for neuropsychiatric disorders.

The anatomy, molecular biology, functions, and regulatory mechanism of hypothalamic kisspeptin–KissR system have been extensively studied, and summarized in a number of review articles for mammalian species (5, 21–23) and for non-mammalian species (10, 24–29). However, the knowledge on non-hypothalamic kisspeptin–KissR system is still limited (14, 30–33). The role of non-hypothalamic kisspeptin signaling is scarcely examined in non-mammalian vertebrates. In this review, we provide an overview and recent updates of non-hypothalamic kisspeptin–KissR systems in non-mammalian vertebrates, with specific emphasis on the habenular Kiss1-KissR1 system in the zebrafish model.

## Two Kisspeptins and KissR Types in Fish

Since their first identification in zebrafish and medaka (9), two kisspeptin types have been identified in several teleost species (24, 25), but some fish species, such as tilapia (34), *Astatotilapia burtoni* (27), red seabream (35), Atlantic halibut (36), flatfish Senegalese sole (37), black rockfish (38), Japanese flounder (39), and puffer fish (40) are likely to possess only one (Kiss2) type. Similar to multiple kisspeptin forms, multiple KissR (KissR1, KissR2, KissR3, and KissR4) types have also been identified in various fish species (25, 28, 41). In zebrafish, there are two KissR types, KissR2 and KissR3 (also designated as KissR1a and KissR1b, respectively) (41, 42). Pharmacological assays verified the ligand-receptor affinities for two kisspeptins and their receptors (28). In zebrafish, while zebrafish Kiss1 peptide (zfKiss1-10) activates KissR3 more efficiently than zebrafish Kiss2 peptide (zfKiss2-10), KissR2 is activated by both zfKiss1-10 and zfKiss2-10 in zebrafish (42). Distribution of two KissR types in the brain further verified the classification of their relationship with two kisspeptin types. In zebrafish, KissR3 gene is widely expressed in the brain, whereas KissR2 gene and its protein product are mainly expressed in the habenula (43–45), where Kiss1 gene is expressed. Therefore, based on these characteristics, we designate zebrafish KissR2 and KissR3 as KissR1 (*kissr1*) and KissR2 (*kissr2*), respectively in our articles (10). However, in some teleost species, relationship between multiple kisspeptins and their receptors has not been clearly characterized because multiple kisspeptins and receptors can cross talk with each other and have different neuroanatomical distributions. Nevertheless, it is very clear that two kisspeptin–KissR types are highly conserved in teleosts species, which are, however, involved in wide range of functions in the brain.

Similar to mammalian species, several functional assays have revealed the major role of Kiss-KissR systems in the control of reproduction in fish. In some fish, including zebrafish, *in vivo* assays show that Kiss2 (Kiss2–10 or Kiss2–12) rather than Kiss1 (Kiss1–10 or Kiss1–15) mainly exhibits its stimulatory effect on gonadotropin synthesis and release (9, 46–48). In chub mackerel, Kiss2 dodecapeptide (Kiss2–12) but not Kiss1 pentadecapeptide (Kiss1–15) administration alters GnRH1, LHβ, and FSHβ genes expression (47), which is further supported by co-expression or proximity of KissR2 in preoptic-hypothalamic GnRH neurons reported in several fish species (6, 49, 50). On the other hand, in some species, such as medaka, Kiss1 seems to be more potent than Kiss2 in the regulation of gonadotropin stimulation. In chub mackerel, Kiss1–15 is more potent than Kiss2–12 on stimulation of gonadal maturation when it was administered chronically (51, 52). In male yellowtail kingfish, Kiss1–10 and Kiss2–10 administration resulted in different effects depends on duration of treatment and reproductive stages of fish (53). These results indicate that regardless of kisspeptin types, fish kisspeptin can stimulate reproductive functions, which, however, may vary dependent on reproductive stages, gender, fish species, and treatment methods.

## Expression of Kiss1–KissR1 in the Teleost Habenula

Expression of Kiss1 gene in the ventral part of the habenula has been shown in the zebrafish (9, 44, 45) as well as in the medaka (9, 54), goldfish (55), European sea bass (56), and the orange spotted grouper (57). However, unlike zebrafish Kiss1 gene expression, in other fish species, Kiss1 gene is also expressed in some brain regions, such as the preoptic-hypothalamic area, suggesting that Kiss1 can act on multiple action sites and have different roles in these species. In contrast to habenular kisspeptin, the expression of habenular KissR1 in the zebrafish, is seen in only a limited fish species. In medaka, KissR1 gene is expressed in the habenula and preoptic nuclei (58). In the European sea bass, not only KissR1 but also KissR2 expression has been reported in the habenula (56). Interestingly, in some species such as the chum mackerel and striped bass, KissR1 gene is expressed in the ventral habenula and preoptic area, in spite of the lack of Kiss1 gene expression in the habenula (50, 59). These results suggest that expression of KissR1 in the habenula is conserved at least among teleosts species that possess two kisspeptin types. In zebrafish, immunohistochemical localization using antibodies specific to zebrafish-Kiss1 and KissR1 reveal that habenular Kiss1/KissR1 cells project to the ventro-anterior corner of the median raphe (vaMR), a subregion of the MR [a division of the superior raphe (SR)] through the fasciculus retroflexus (FR) (44, 45, 60), which has also been confirmed in a *kiss1*:mCherry transgenic zebrafish (61). However, in the zebrafish brain, the KissR1 antibody also labeled cells in other brain area, such as the telencephalon, diencephalon, and spinal cord regions (60). This is because of the cross-reactivity of the KissR1 antibody against kissr1b-derived protein 2, an alternative splice variant of the KissR1 gene, which shares the epitope of the KissR1 antibody (62). Zebrafish KissR1 gene possess four additional alternative splice variants encoding different protein lengths (KRBDP 1–4), which, however, are functionally incapable of mediating kisspeptin-derived cellular responses (62). In the zebrafish, Kiss1 and its receptor are co-expressed in the same neurons within the habenula (63). Furthermore, central administration of Kiss1 peptides significantly suppresses Kiss1 gene expression, suggesting an autocrine regulation of the Kiss1 gene (63).

## Modulation of Serotonin and its Related Behaviors by Habenular Kiss1/KissR1

Habenular Kiss1/KissR1 cells send projections in the vicinity of serotonin [5-hydroxytryptamine (5-HT)]-containing neurons located the median raphe (60). Mammalian habenula consists of two major subnuclei, the medial and lateral habenula. The medial habenula projects to the interpeduncular nucleus (IPN), while the lateral habenula directly projects to the ventral tegmental area and raphe, which are dopaminergic and serotonergic structures, respectively (64). Similar to mammalian habenula, the fish habenula can also be neuroanatomically subdivided into two major nuclei, the dorsal and ventral habenula based on difference in their cytoarchitectual structures (65). In zebrafish, the dorsal habenula project to the IPN *via* FR, while the ventral habenula project to the vaMR (60, 66). In addition, the dorsal habenula express the POU-domain transcription factor *brn3a*, a marker for the mice medial habenula (67, 68), while the ventral habenula express *protocadherin 10a*, a specific marker of the rat lateral habenula (66, 69). Therefore, the fish dorsal and ventral habenula have been elucidated as the homolog of the mammalian medial and lateral habenula, respectively. In mammals, the lateral habenula has been implicated as a pivotal regulator of dopaminergic and serotonergic neurons (19, 70). Furthermore, the lateral habenula is involved in sleep, locomotion, motivation, reward, and behavioral stress responses (19). In the brain of zebrafish treated with zfKiss1–15 peptides, expression of genes associated with serotonin, *pet1* and *sert* (*slc6a4a*), and *c-fos* genes are significantly upregulated within the raphe nucleus (63). Central administration of zfKiss1–15 has no effect on anxiety, but shows a trend in anxiolytic effect (increase in number of transition) in zebrafish (45) when observed using a novel-tank diving test (71). Fish administered with zfKiss1–15 peptides failed to exhibit fear (45), characterized by erratic and freezing behaviors in response to an aversive stimulus from skin extract (alarm substance) (72, 73). Such effects were not observed when zfKiss2–10 was administered, suggesting these effects could be mainly modulated by Kiss1–KissR1 pathway. In addition, injections of zfKiss1–15 peptides conjugated with saporin; a ribosome-inactivating cytotoxic protein (74) induced cell death of Kiss1 neurons, the immunoreactivity of KissR1 was significantly reduced in the habenula and median raphe, and in these fish, alarm substance-induced fear response was significantly reduced (45). A recent study using *kiss1*-mutant fish revealed the potential involvement of habenular Kiss1 neurons to avoid punishment (75). These Kiss1 gene*-*mutants have a stop codon upstream of the active Kiss1 peptide, which causes deficiency in learning to avoid a shock that is predicted by light. These studies suggest that Kiss1–KissR1 signaling modulates behavioral response to uncontrollable aversive stimuli in the habenula. However, possible involvement of KissR2 or other receptor signaling pathways in this behavioral response should not be ignored, because KissR2 or other GPCRs are also activated by both Kiss1–10 and Kiss2–10 (42, 76).

## Mechanism of Serotonergic Modulation by Kiss1–KissR1 Signaling

**Figure 1:**
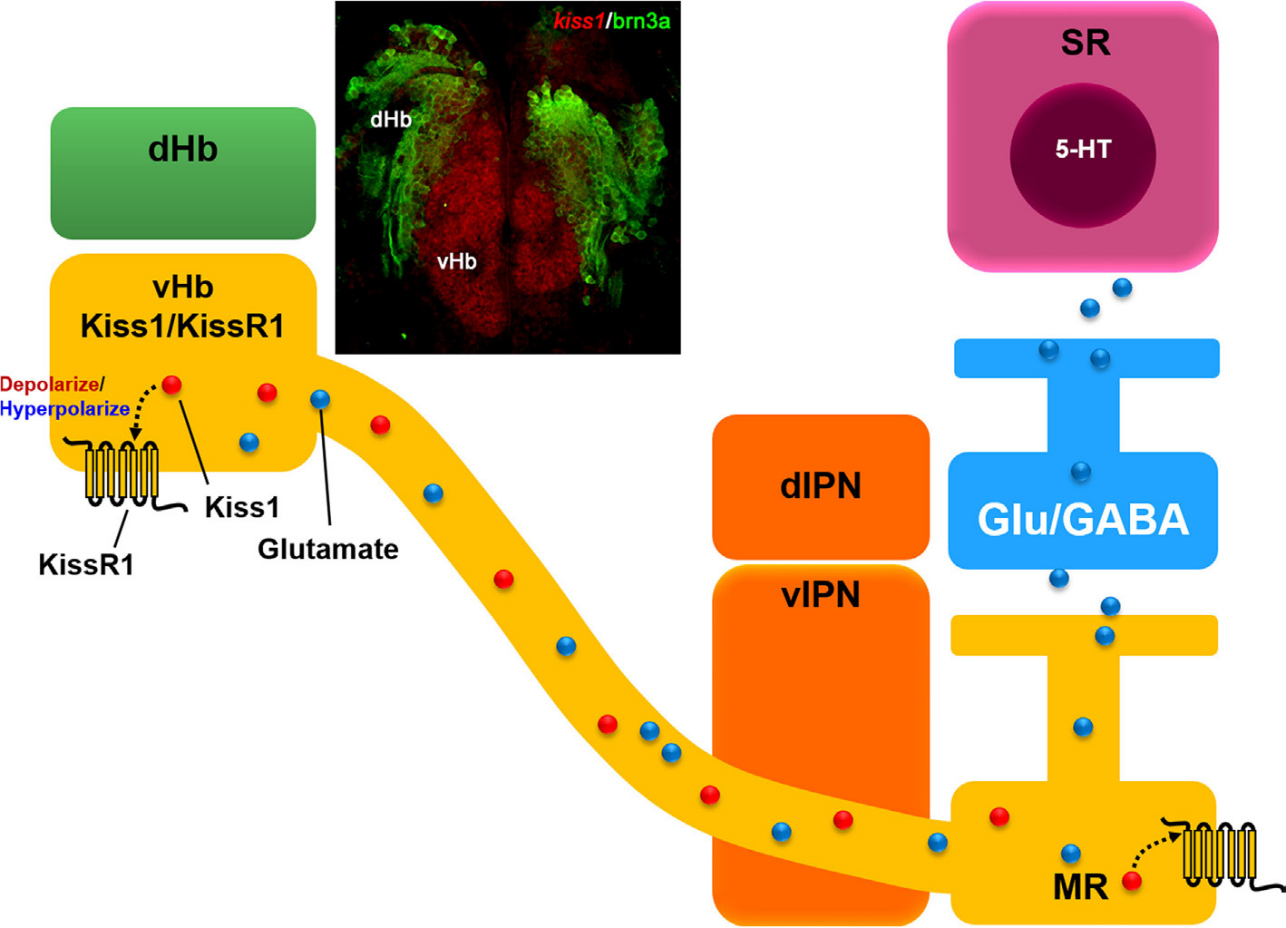
Schematic drawing of hypothetical neural circuit of habenular kisspeptin 1 (Kiss1) neurons. Kiss1 (red dot) modulate ventral habenular (vHb) neuronal activities *via* concentration-dependent mechanism through co-expressing Kiss1 receptor (KissR1). Photomicrograph shows a coronal brain section image of *kiss1* mRNA expression in the vHb (red) but not in the dorsal habenula (dHb, green) expressing *brn3a*, a marker gene for the dHb in transgenic (*brn3a-hsp70:GFP*) zebrafish. Habenular Kiss1 neurons send their projections to the median raphe (MR), a division of the superior raphe (SR). Kiss1 cells are glutamatergic in nature and it is hypothesized that the presynaptic action of the Kiss1/KissR1 system causes the release of glutamate (blue dot) in Kiss1 cells from the vHb that potentially regulates the serotonin (5-HT) system in the SR directly or *via* glutamatergic and GABAergic neurotransmission. Abbreviations: dIPN, dorsal interpeduncular nucleus; vIPN, ventral interpeduncular nucleus modified from Nathan et al. (60).

zfKiss1–15 administration effects serotonin-related genes, although KissR1 is not expressed in serotonergic neurons (60, 63), indicating that Kiss1 neurons act indirectly through interneurons on serotonergic system in zebrafish. Habenular Kiss1 neurons are glutamatergic in nature and their axons form close association with either glutamatergic or GABAergic interneurons in the median raphe region (60) (Figure 1). This suggests that Kiss1 might regulate serotonergic neural activities *via* the modulation of glutamatergic presynaptic neurotransmission. We speculate KissR1 might function as a presynaptic autoreceptor on habenula Kiss1 nerve terminals to facilitate glutamatergic transmission on serotonergic neurons, which remains to be confirmed.

In the zebrafish, although Kiss1 inhibits alarm substance-induced fear response, but its involvement through the serotonergic system remained unclear. The effect of Kiss1 on alarm substance-induced fear responses was blocked in the presence of serotonin receptor antagonists (77). Interestingly, treatment with different antagonist against two serotonin receptor types (5-HT_1A_ and 5-HT_2_) results in different behavioral responses. Anxiolytic effect of Kiss1 is modulated *via* 5-HT_1A_ receptor, while inhibitory effect of Kiss1 on freezing behavior is modulated *via* 5-HT_2_ receptor (77). Further, calcium imaging study has shown that Kiss1-gene mutant zebrafish larvae have reduced activation of raphe neurons by aversive stimulus (75). These studies indicate that Kiss1–KissR1 signaling is involved in the modulation of serotonergic neural activity under uncontrollable aversive conditions. Administration of exogenous Kiss1 or ablations of Kiss1 neurons suppresses alarm substance-induced fear response (45). Surprisingly, administration of Kiss1 also elevates *c-fos* gene expression by cellular excitation in the ventral habenula neurons (63). Optogenetic stimulation of ventral habenula neurons evokes place avoidance behavior (78), which is contradictory to the results of *c-fos* expression. This issue has been recently resolved by an electrophysiological approach (75), where Kiss1 has been shown to have a concentration-dependent effect on ventral habenula neurons: depolarizing at low concentrations and hyperpolarizing at high concentrations. Furthermore, *c-fos* expression was induced by a concentration of 10^−11^ mol/fish of Kiss1 peptides, but not with a higher concentration of 10^−9^ mol/fish (63). Therefore, suppression of alarm response by exogenous Kiss1 peptides could be due to hyperpolarization of ventral habenula neurons, which, however, remains to be further examined by functional assays.

Although Kiss1–KissR1 signaling modulates serotonin in response to uncontrolled aversive stimulus, but it remains unclear what regulates Kiss1 neurons, including Kiss1 peptide synthesis and secretion and Kiss1 neuronal activities. More importantly, the role of Kiss1–KissR1 signaling within the habenula neurons is still unknown. It is also important to identify the upstream control of the ventral habenula neurons, which could be from several afferent projections from brain regions, such as the entopeduncular nucleus, preoptic area, and hypothalamus (79, 80). Recent studies have revealed a functional connection between a thalamic nucleus and the habenula in zebrafish, and this pathway mediates light-evoked locomotor activity, including circadian behavior (81, 82). In mammals, the habenula (lateral habenula) neurons have been suggested to act as circadian oscillators (83–85). In addition, in the goldfish, Kiss1 and KissR gene expression are influenced by different light spectra (86). Therefore, Kiss1–KissR1 signaling in the habenula could be involved in mediating circadian controlled innate behaviors, such as sexual behavior and sleep–wake cycle, which remain to be studied.

## Biological Significance of Habenular Kisspeptin

In a series of our studies, we have provided some evidences for the involvement of kisspeptin–KissR signaling in the zebrafish vertebrate habenula (45, 60, 63, 77). However, the presence of Kiss1 in the fish habenula nuclei has been shown in a limited number of fish species. Some fish species possess only one type of kisspeptin gene (Kiss2), which is expressed in the hypothalamic area (10). Some fish species that possess two kisspeptin types such as the chum mackerel show no expression of Kiss1 in the habenula (59). On the other hand, expression of KissR in the habenula has been identified in several fish species and also in mammals (11, 87) (Table 1) and kisspeptin neurons in the anteroventral periventricular nucleus have been shown to innervate the habenula (88), suggesting that the action of kisspeptin–KissR signaling and its role within the habenula might be evolutionarily conserved regardless of the source of kisspeptin. Interestingly, in the fetal mouse brain, Kiss1R containing cells are seen in a highly restricted population of cells in the habenula as early as embryonic day 17 (89), which is a period when the formation of habenula-IPN pathway is completing (90). Similarly, in embryonic zebrafish, Kiss1-positive cells are first appearing in the ventral habenula by 5-days post fertilization, when the innervation of the ventral habenula axon has reached their target, the medial raphe (91). Furthermore, in a mutant fish that lacks functional Tcf7l2, a downstream modulator of the Wnt signaling cascade, Kiss1 gene expression is lost and the median raphe are not innervated by ventral habenula axons (91). Therefore, the habenular Kiss1–KissR1 signaling may play a role in the habenula axonal formation during brain development. Previous studies have demonstrated the role of the lateral habenula in the hormonal onset of maternal behavior in female rats (92, 93). In both rodent and fish, the habenula is known to be sensitive to steroid hormones and express estrogen receptors (94, 95). In addition, the habenula in the zebrafish produces neurosteroids locally (96, 97). Furthermore, goldfish Kiss1 gene promoter contains putative binding sites for estrogen receptors (55), and in orange-spotted grouper, Kiss1 neurons express estrogen receptors in the habenula (57). Estrogen has effect on mood, mental state, and memory, which are closely related to serotonergic functions (98). Interestingly, in larval zebrafish, treatment with an estrogen receptor β agonist increased *c-fos* expression in the habenula with anxiolytic effect (increase in exploration behavior) (95). Therefore, kisspeptin–KissR signaling pathway within the habenula could be involved in the neuromodulatory processes of emotional and goal-directed behaviors, which could also be influenced by reproductive conditions.

**Table 1:** Kisspeptin and kisspeptin receptor (KissR) types and their expression in the habenula.

Species	Kisspeptin types	Expression (cell body) in the habenula	KissR types	Expression in the habenula	Reference
Rat	Kisspeptin 1 (Kiss1)	−	Kiss1R	+	(11)
Mouse	Kiss1	−	Kiss1R	+	(13, 87, 89)
Syrian hamster	Kiss1	−	Kiss1R	+	(99)
*Xenopus laevis*	Kiss1 Kiss2	− −	GPR54-1a GPR54-1b GPR54-2	ND ND ND	(42)
Zebrafish (*Danio rerio*)	Kiss1 Kiss2	+ −	Kiss1 receptor (KissR1) KissR2	+ −	(9, 44, 63)
Medaka (*Oryzias latipes*)	Kiss1 Kiss2	+ −	KissR1 KissR2	+ −	(9, 54, 58)
Goldfish (*Carassius auratus*)	Kiss1 Kiss2	+ −	KissR1 KissR2	ND ND	(55)
Striped bass (*Morone saxatilis*)	Kiss1 Kiss2	− −	KissR1 KissR2	+ −	(50)
European sea bass (*Dicentrarchus labrax*)	Kiss1 Kiss2	+ −	KissR1 KissR2	+ +	(56)
Orange-spotted grouper (*Epinephelus coioides*)	Kiss1 Kiss2	+ −	KissR1 KissR2	ND ND	(57)
Chum mackerel (*Scomber japonicas*)	Kiss1 Kiss2	− −	KissR1 KissR2	+ −	(59)
Sapphire devil (*Chrysiptera cyanea*)	Kiss1 Kiss2	+ −	KissR1 KissR2	ND ND	(100)
Nile tilapia (*Oreochromis niloticus*)	Kiss2	−	KissR2	ND	(34)
*Astatotilapia burtoni*	Kiss2	ND	KissR2	+	(49)
Red seabream (*Pagrus major*)	Kiss2	−	NA	ND	(35)
Grass puffer (*Takifugu niphobles*)	Kiss2	−	KissR2	ND	(101)

*+, confirmed expression of kisspeptin and KissR types in the habenula; −, confirmed lack of expression of kisspeptin and KissR types in the habenula; ND, expression of kisspeptin and KissR types in the habenula has not been determined*.

## Author Contributions

Both authors researched and wrote/edited the article and designed the figures.

## Conflict of Interest Statement

The authors declare that the research was conducted in the absence of any commercial or financial relationships that could be construed as a potential conflict of interest.
